# Supplementary material for: Exploring Large Protein Sequence Space through Homology- and Representation-based Hierarchical Clustering
Source: Mol Biol Evol. 2025 Jun 4;42(6):msaf136. doi: 10.1093/molbev/msaf136 (PMC12204192; doi:10.1093/molbev/msaf136)
Supplement: msaf136_Supplementary_Data [file msaf136_supplementary_data.zip › MBE supplementary.pdf]

Supplementary Information:

Exploring large protein sequence space through homology- and representation-based hierarchical clustering

John Z. Chen [1,2], Barnabas Gall [1,3], Sacha B. Pulsford [1,3], Nobuhiko Tokuriki [4], Colin J. Jackson [1,2,3,5]

1. Research School of Chemistry, Australian National University, Canberra, Australia
2. ARC Centre of Excellence in Synthetic Biology, Research School of Biology, Australian National University, Canberra, ACT 2601, Australia
3. ARC Centre for Innovations in Peptide & Protein Science, Research School of Chemistry, Australian National University, Canberra, ACT 2601, Australia
4. Michael Smith Laboratories, University of British Columbia, Vancouver, Canada
5. Research School of Biology, Australian National University, Canberra, ACT 2601, Australia

Corresponding: John Z. Chen, john.chen@anu.edu.au

Supplementary Figures

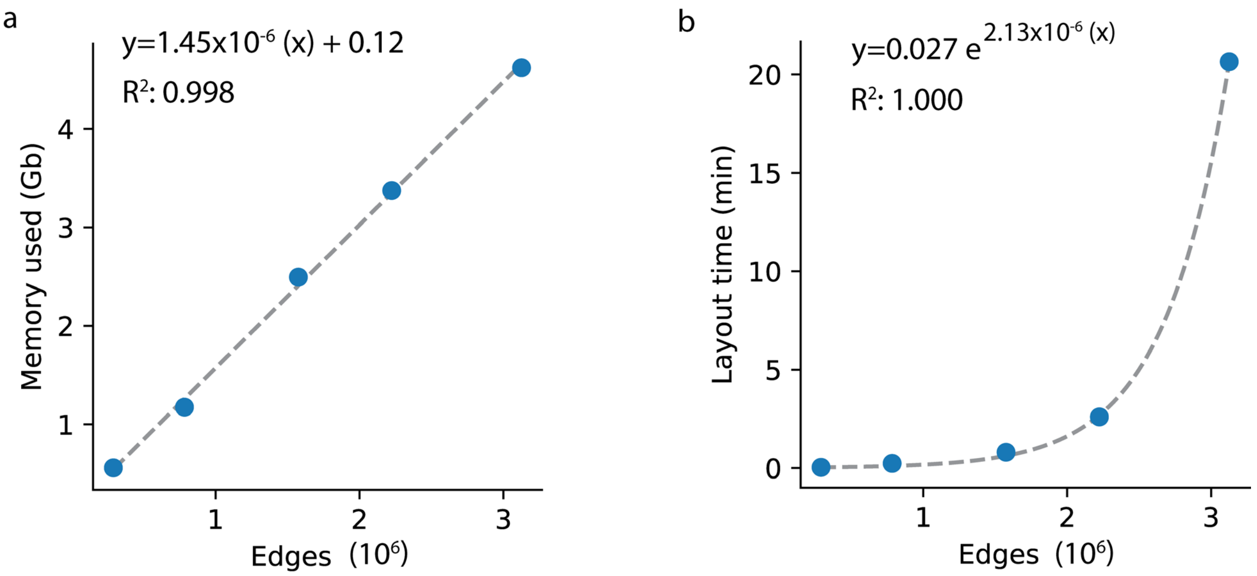

Supplementary Figure 1. Resource use in Cytoscape as a function of the number of edges in the network. The FMN-binding split barrel superfamily was divided into single networks at bitscore cut-offs of 500-900 in 100 bitscore steps, and time and memory used for visualization in Cytoscape was measured and used to calculate resource use with edge count. (**a**) Gb of memory used for networks of given edge count, fitted to a linear regression. (**b**) Time to generate default layout using Perfuse force directed algorithm for networks of given edge count, fitted to an exponential function. Lines of best fit, equations of the fit and R^2 are shown in the plot.

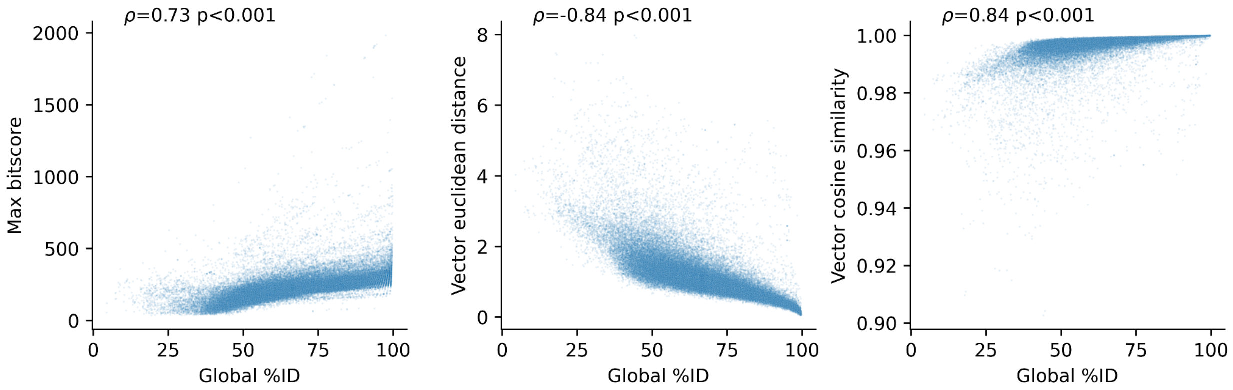

Supplementary Figure 2. Correlation of sequence similarity metric to global sequence identity. The data was a sample of 71,006 pairs of sequences (excluding self pairing). Max bitscore is calculated from pairwise BLAST between sequences, taking the max value in the case of reciprocal pairs. Vector representations are compared by either their euclidean distance or cosine similarity. The spearman correlation coefficient and p-value are displayed.

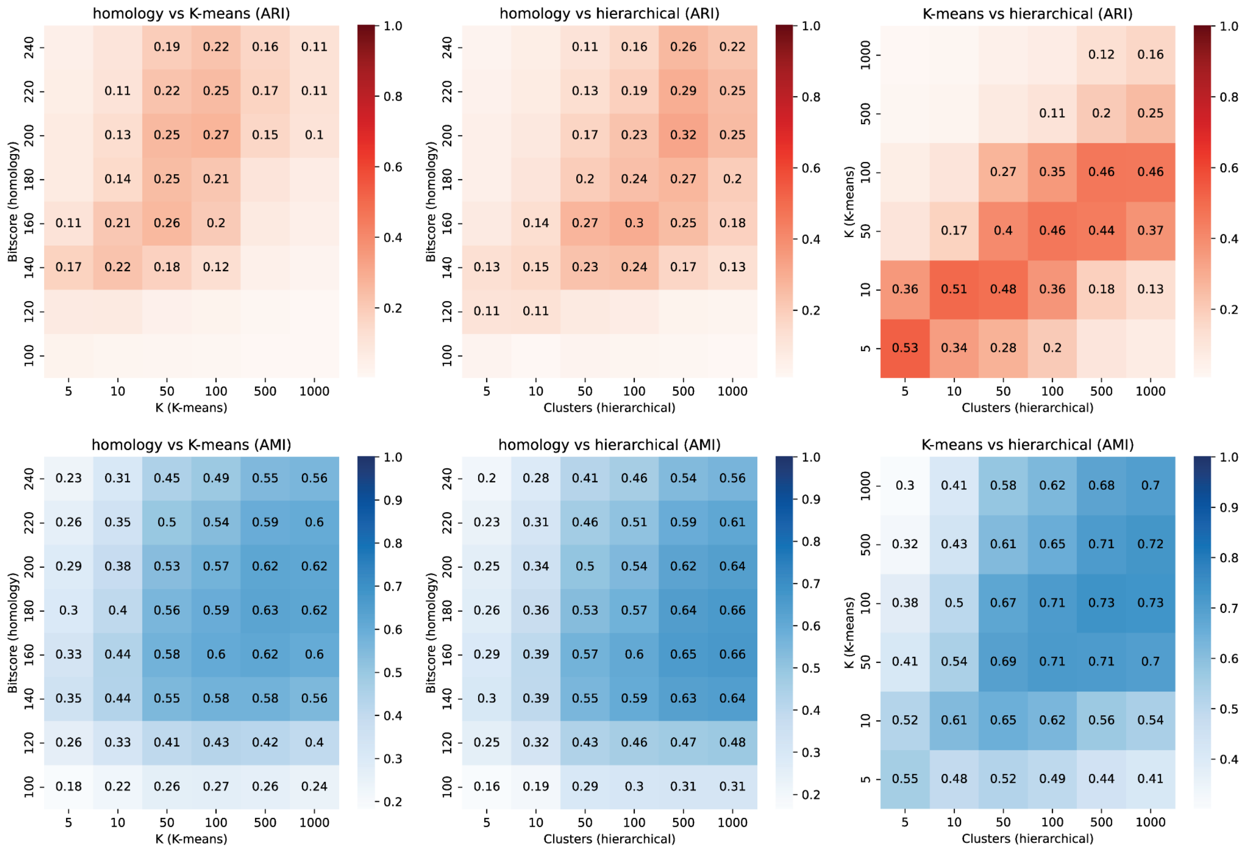

Supplementary Figure 3. Measure of agreement between cluster assignments between each pair of clustering methods. Heatmaps show the clustering agreement, as measured by the adjusted Rand index (ARI) or the adjusted mutual information (AMI) between each pair of cluster definitions. The clusterings are arranged by the bitscore cut-off for homology, while K-means and hierarchical clustering are arranged by the number of target clusters.

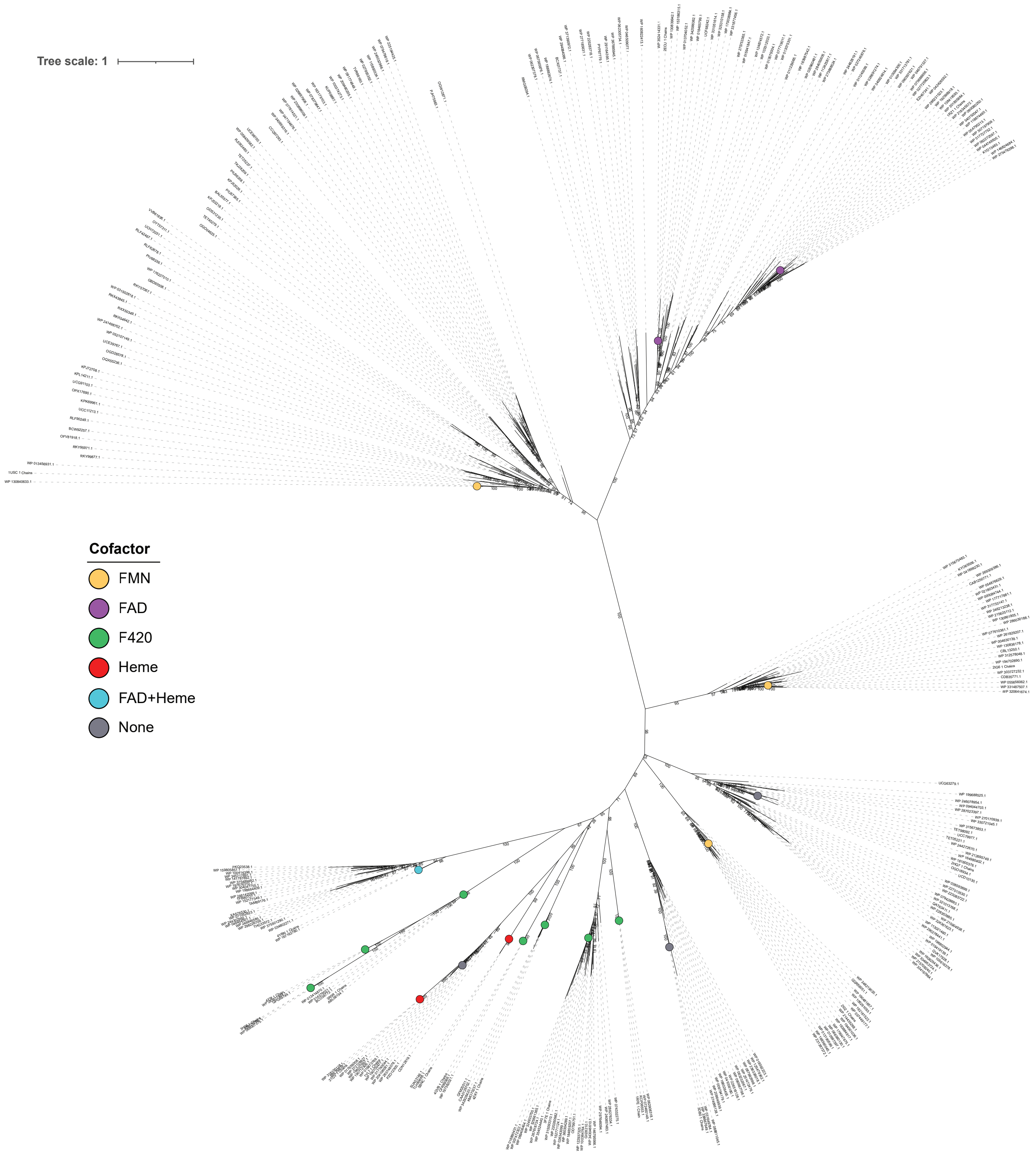

Supplementary Figure 4. BLAST sampling based maximum likelihood tree of FMN-binding split barrel superfamily. The tree contains 321 leaves. The 18 structural representatives and their co-factors are highlighted. Bootstrap values are displayed as numbers.

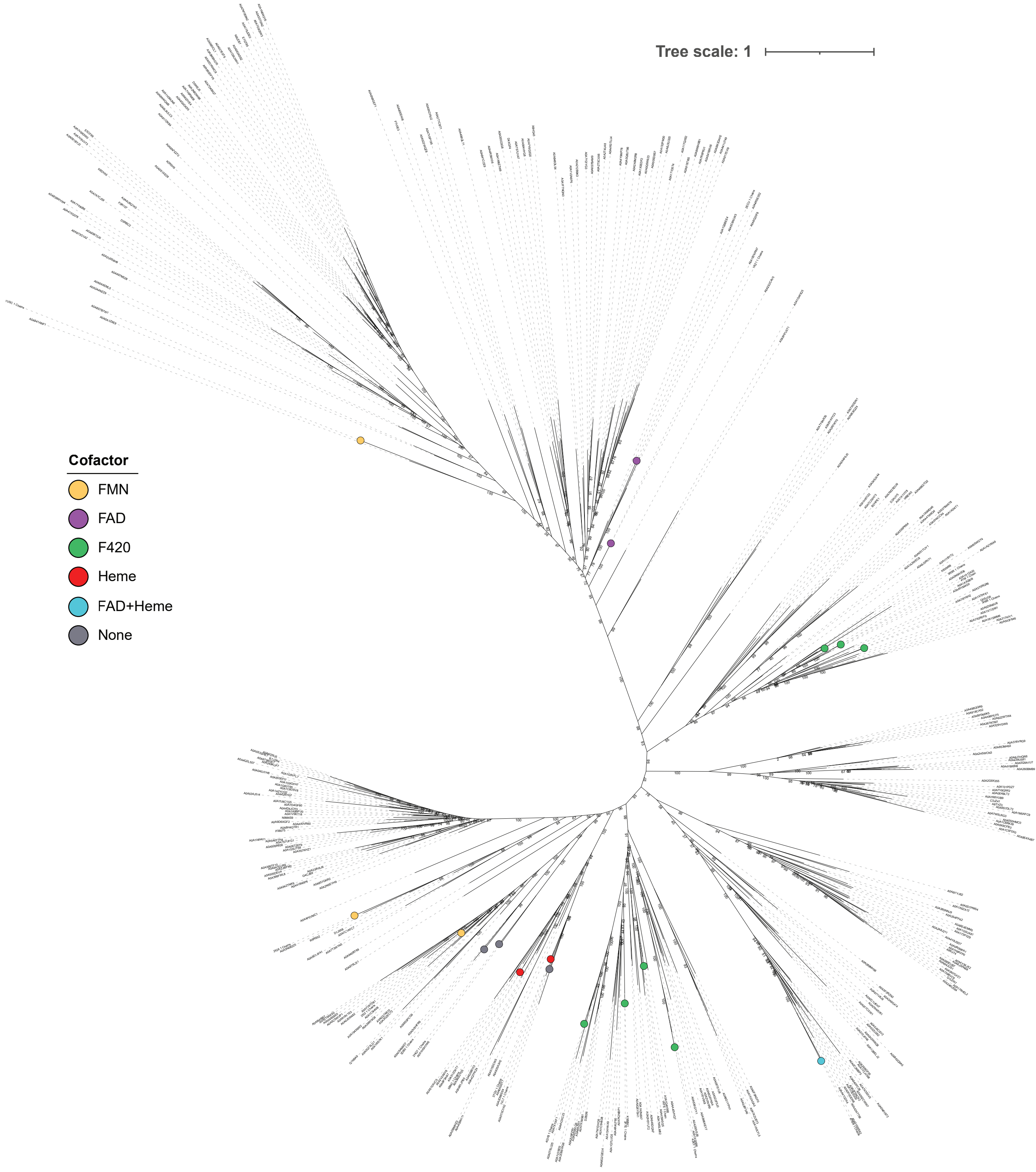

Supplementary Figure 5. Cluster tools sampling based maximum likelihood tree of FMN-binding split barrel superfamily. The tree contains 364 leaves. The 18 structural representatives and their co-factors are highlighted. Bootstrap values are displayed as numbers.

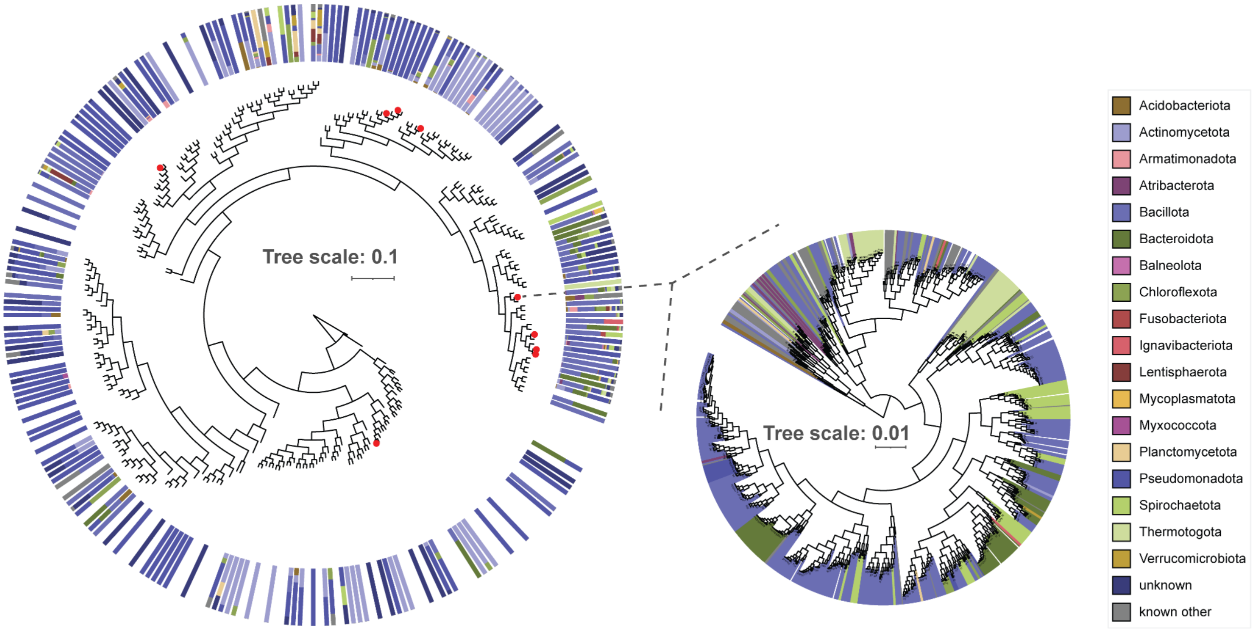

Supplementary figure 6. Tree structure visualization of hierarchical clustering results. The tree structure of the Periplasmic binding protein/LacI sugar binding domain (IPR001761). The full tree structure of ~40k sequences was constructed using the hierarchical clustering method on the sequence vector representations. The full tree was condensed to 300 clusters (left) and annotated according to taxonomy, leaves highlighted in red have SwissProt annotations. The smaller tree on the right illustrates the annotated subtree from one of the clusters.

Supplementary Tables

Supplementary Table 1. Observed resource usage for network visualization in Cytoscape

Bitscore	Edges (10^6)	Memory (Gb) [a]	Layout Time (min) [b]	Connected sequences [c]	Percent connected [d]
900	0.3	0.6	0.03	6,001	2%
800	0.8	1.2	0.2	7,209	2%
700	1.6	2.5	0.8	8,838	3%
600	2.2	3.4	2.6	13,201	4%
500	3.1	4.6	21	25,837	9%

a. Memory usage as reported by Cytoscape after layout is completed and unused memory is manually freed.
b. Time for Cytoscape to generate the default layout using Perfuse force directed algorithm.
c. Number of unique sequences connected to any other non-self sequence.
d. Connected sequences as a percentage of total number of sequences (299,624).

Supplementary Table 2. Estimated resource usage in Cytoscape for network of a given size

Bitscore	Edges (10^6)	Memory (Gb) [a]	Layout Time (M yrs) [b]	Connected sequences [c]	Percent connected [d]
72	251	365	1.0E+213	298,976	100%
102	210	305	9.7E+174	297,077	99%
150	140	203	1.6E+110	290,075	97%
213	70	102	3.8E+45	264,922	88%
279	28	41	3.9E+06	202,711	68%
300	21	30	1.0E+00	175,344	59%
400	6.2	9	10.6 (days)	78,037	26%

a. Estimated memory usage including visualization. Calculated as memory=1.45×10^{-6} (edges) + 0.12, based on linear regression of observed edge count to memory usage.
b. Estimated time for Cytoscape to generate the default layout using Perfuse force directed algorithm. Calculated as time=$0.027e^{(2.13 \times 10^{-6})(edges)}$, based on exponential fit of observed edge count to layout time.
c. Number of unique sequences connected to any other non-self sequence.
d. Connected sequences as a percentage of total number of sequences (299,624).
